# Louisville Summer School of Medicine

**Published:** 1844-10

**Authors:** 


### LOUISVILLE SUMMER SCHOOL OF MEDICINE.

It gives us pleasure to announce that the enterprise of conducting a course of medical instruction in Louisville during the vacation in the Medical Institute has been so far successful, that it is the determination of a number of the gentlemen engaged in it to prosecute it next summer. Students of medicine, therefore, coming to the Institute this fall, would do well to come prepared to pass the vacation in the city, where, in addition to the fine library of the Institute, and the advantages offered in the summer course of lectures, the most desirable facilities are afforded by the Hospital for the study of clinical medicine. The hospital for months past has been filled with patients, and constitutes, during the sickly season, one of the best schools for the study of disease.

